# Design of a multi-epitope-based peptide vaccine against the S and N proteins of SARS-COV-2 using immunoinformatics approach

**DOI:** 10.1186/s43042-022-00224-w

**Published:** 2022-02-04

**Authors:** Arian Karimi Rouzbahani, Farnaz Kheirandish, Seyedeh Zeinab Hosseini

**Affiliations:** 1 Student Research Committee, Lorestan University of Medical Sciences, Khorramabad, Iran; 2 Department of Medical Parasitology and Mycology, School of Medicine, Lorestan University of Medical Sciences, Khorramabad, Iran; 3 Department of Medical Biotechnology, School of Medicine, Lorestan University of Medical Sciences, Khorramabad, Iran; 4 Razi Herbal Medicines Research Center, Lorestan University of Medical Sciences, Khorramabad, Iran

**Keywords:** SARS-CoV-2, Multi-epitope, Vaccine, Immunoinformatics, Antigenicity

## Abstract

**Background:**

As the new pandemic created by COVID-19 virus created the need of rapid acquisition of a suitable vaccine against SARS-CoV-2 to develop Immunity and to reduce the mortality, the aim of this study was to identify SARS-CoV-2 S protein and N antigenic epitopes by using immunoinformatic methods to design a vaccine against SARS-CoV-2, for which S and N protein-dependent epitopes are predicted. B cell, CTL and HTL were determined based on antigenicity, allergenicity and toxicity that were non-allergenic, non-toxic, and antigenic and were selected for the design of a multi-epitope vaccine structure. Then, in order to increase the safety of Hbd-3 and Hbd-2 as adjuvants, they were connected to the N and C terminals of the vaccine construct, respectively, with a linker. The three-dimensional structure of the structure was predicted and optimized, and its quality was evaluated. The vaccine construct was ligated to MHCI. Finally, after optimizing the codon to increase expression in *E. coli* K12, the vaccine construct was cloned into pET28a (+) vector.

**Results:**

Epitopes which were used in our survey were based on non-allergenic, non-toxic and antigenic. Therefore, 543-amino-acid-long multi-epitope vaccine formation was invented through linking 9 cytotoxic CTL, 5 HTL and 14 B cell epitopes with appropriate adjuvants and connectors that can control the SARS coronavirus 2 infection and could be more assessed in medical scientific researches.

**Conclusion:**

We believe that the proposed multi-epitope vaccine can effectively evoke an immune response toward SARS-CoV-2.

**Supplementary Information:**

The online version contains supplementary material available at 10.1186/s43042-022-00224-w.

## Background

Since the advent of SARS-CoV in 2002 and its spread to 32 countries, the world has experienced the outbreak of MERS-CoV and now nCoV 2019 [1]. Coronavirus 2019 (COVID-19), caused by SARS-CoV-2, was first reported in a number of patients with pneumonia of unknown etiology in Hubei Province, China, and subsequently in many parts of the world [2]. Coronaviruses have four genera: alpha, beta, delta and gamma virus. SARS-CoV-2 belongs to the beta-coronavirus genus with an envelope with a single-stranded RNA genome, positive sense, and has a diameter of about 80–120 nm [3, 4]. Their genome size is about 26–32 kilobases [4]. Coronaviruses can infect humans and other vertebrates and cause infections in the respiratory system, gastrointestinal tract and central nervous system of humans, livestock, birds, bats and mice and many other wild animals [5]. SARS-CoV-2, like other coronaviruses, encodes several structural proteins. The structural proteins of SARS-CoV-2 include nucleoprotein (N), membrane (M), surface glycoprotein (S) and envelop protein (E) [4]. Most coronaviruses require structural protein to produce a complete viral particle [6]. Each of these proteins is not only involved in the structure of the virus but also in various aspects, involved in virus replication [7]. Surface glycoprotein (S) is responsible for binding to the cellular receptor [8], which has two basic components (S1) and globular (S2). S1 is responsible for binding to the cellular receptor, and S2 contains fusion peptide [9]. For SARS-COV, full-length and the active immunization part of S protein [10], S protein peptides [11] and chimeric versions of S protein have been identified [12]. DNA structures encoding the S protein have also produced virus-neutralizing antibodies [13]. S protein as a major antigenic component is an important target for vaccine development [14]. Nucleoprotein (N) is a phosphoprotein and nucleocapsid protein that binds to genomic RNA and M protein and is the main stimulus of the host immune system during viral infection [15]. N protein in its entirety is highly immunogenic and antigenic [16]. In addition, N protein is an early diagnostic marker for SARS-COV because it can be detected in clinical specimens one day after the onset of symptoms [17] and it is stable due to very small mutations [18]. Although ritonavir and lopinavir are used as protease-inhibiting drugs for the treatment of SARS-COV-2, it has been reported in a clinical trial that its usefulness for the treatment of SARS-COV-2 is questionable [19]. In case of emergency, Remdesivir is used against SARS-COV-2 or recovered patients’ plasma is used as a side-effect-free treatment [20]. However, there is no specific and approved drug for SARS-COV-2 infection, and the treatment approach is more supportive, and the use of these therapies is said to reduce the resulting mortality rate. Therefore, the development of effective drugs and vaccines against the control of emerging diseases is a priority of research and immunoinformatic is currently considered as a new method to find an effective way to control diseases [21]. Immunoinformatics methods could be used to explore antigens of viruses, prediction of their epitopes and evaluation of its immunogenicity [22]. In different studies, medical procedures against the Middle East respiratory syndrome coronavirus (MERS-CoV), Zika virus and Ebola virus were performed by utilizing immunoinformatics techniques [23, 24].

The use of epitope vaccines using immunogenic epitopes specific to CD8+ and CD4+ cells and stimulating the immune system against these epitopes simultaneously and completely specifically are among the methods that have been considered in this regard. Conventional methods for producing vaccines are time-consuming and expensive [25]. The immune system can respond to any viral or microbial contamination, by detecting foreign intruders through their artificial peptide epitopes. By having a total map of virus epitopes and their immunogenicity, it is vital to create an effective vaccine against COVID-19 virus disease [26]. Moreover, multi-epitope vaccine significantly stimulates humoral and cellular immune responses, concurrently due to T cell as well as B cell epitopes [27, 28]. Multi-epitope vaccine is made of adjuvants, so they are expected to create long-standing immune reactions and high immunogenicity [28].

The aim of this study was to evaluate T-cell- and B-cell-dependent epitopes derived from SARS-CoV-2 S and N antigens for the design and development of a multi-epitope vaccine based on the analysis of immunoinformatic tools.

## Methods

### Sequence extraction and protein structure

The FASTA sequences of S protein (YP_009724390.1) and N protein (YP_009724397.2) SARA-COV-2 were retrieved from the NCBI GenBank database (https://ww.ncbi.nlm.nih.gov/) and also Human β-defensin-2 (PDB ID: 1FD3) and 3 (PDB ID: 1KJ6) from PDB database (https://www.rcsb.org).

### Prediction of B cell immune epitopes

An antigen must be able to elicit both the B and T cell immune responses in order to be a suitable candidate for the vaccine. Therefore, for predicting B cell linear epitopes two servers were used, IEDB (https://www.iedb.org/) and ABCPred (http://crdd.osdd.net/raghava/abcpred/ABC_submission.html). ABCPred ranks epitopes by using ANN scores, according to the score obtained and above the threshold (0.5); thus, it is more probable for a sequence to be an epitope with a higher score. For predicting linear epitopes with ABCPred server, 16 mer length of epitopes with default threshold (0.51) and for IEDB server with BepiPred linear epitope prediction method which predicts the location of B cell linear epitopes using a combination of a hidden Markov model and an orientation degree method were selected by default [29]. From matching the predicted linear epitopes with the two servers of IEDB and ABCPred, the epitopes with the highest overlap were selected for further study.

### Prediction of CTL and HTL epitopes

In order to predict CTL epitopes, ComPred method (combination of artificial neural network method and quantitative matrix) was used along with default cutoff score (0.5) of nHLAPred server (https://webs.iiitd.edu.in/raghava/nhlapred.comp.html). 0.18 epitopes were selected with the highest score of alleles and with the highest frequency of Iranian population (HLA * A02:01) and (HLA * B35:01) according to the server (http://allelefrequencies.net/hla6006a.asp) for analyzing the next ones. HTL epitopes for DRB1 * 0101, DRB1 * 1101 and DRB1 * 1501 alleles (alleles with the highest frequency from the population of Iran) from NetMHCIIpan 4.0 server (http://www.cbs.dtu.dk/services/NetMHC) were determined [30]. The default threshold was considered for strong connections (rank 0.5%), weak connections (rank 2%) and prediction epitope of 15 amino acids long. Predicted epitopes with strong connections were used for further studies.

### Evaluation of B cell, CTL and HTL epitopes based on allergenicity, antigenicity and toxicity parameters

Since the components of the vaccine must be capable of allergic reactions, the selected epitopes for B cell, CTL and HTL with the AllerTOP server v. 2.0 (https://www.ddg-pharmfac.net/AllerTOP/index.html) were reviewed to ensure the ability of selected epitopes to induce an immune response with the VaxiJen v. 2.0 server (http://www.ddg-pharmfac.net/vaxijen/VaxiJen/VaxiJen.html) and were examined with a threshold of 0.4. This is because toxic epitopes can compromise the structure of the vaccine and should be removed. ToxinPred server (https://webs.iiitd.edu.in/raghava/toxinpred/design.php) with SVM method and default server parameters was used to determine toxic epitopes. Finally, antigen, non-allergenic and non-toxic epitopes were selected as possible epitopes for CTL and HTL B cell.

### Vaccine structure design

The epitopes which were chosen in the previous steps for CTL, HTL and B cell, were selected to design the vaccine structure and were connected by AAY, GPGPG and KK linkers, respectively. In order to improve the immune response, hBD-3 connected to the N terminal and hBD-2 connected to the C terminal of the vaccine construct as an adjuvant to the EAAAK linker.

### Evaluation of allergenicity, antigenicity, solubility and stereochemical properties of vaccine structures

Allergenicity assessment has the ability to predict the structure of the vaccine in causing allergies and allergic reactions. Accordingly, the Allergen FP 1.0 server (http://ddg-pharmfac.net/AllergenFP/) [31] was used. Structural antigenicity designed with ANTIGENpro server (http://imed.med.ucm.es/Tools/antigenic.pl) [32] and VaxiJen v2.0 (http://www.ddg-pharmfac.net/vaxijen/VaxiJen/VaxiJen.html) [33] was examined. Vaccine construct solubility prediction was performed with SOLpro server (http://scratch.proteomics.ics.uci.edu/) [34]. The ProtParam server (https://web.expasy.org/protparam) [35] was used to predict stereochemical properties.

### Predicting the second and third structures

SOPMA server (https://npsa-prabi.ibcp.fr/cgi-bin/npsa_automat.pl?page=/NPSA/npsa_sopma.html) and the PSIPRED server (http://bioinf.cs.ucl.ac.uk/psipred/) were used to identify the second structure of the vaccine construct, and the phyre2 server (http://www.sbg.bio.ic.ac.uk/~phyre2/html/page.cgi?id=index), RaptorX server (http://raptorx.uchicago.edu/ContactMap/) and I-TASSER (https://zhanglab.ccmb.med.umich.edu/I-TASSER//) were used to predict the third structure of the vaccine.

### Energy optimization and validation evaluation of the third structure of the vaccine structure

To identify and correct the errors of the selected 3D model, the 3D structure was optimized by using the GalaxyRefine server (http://galaxy.seoklab.org/cgi-bin/submit.cgi?type=REFINE). ProSA, ERRAT and Ramachandran servers in the software (https://servicesn.mbi.ucla.edu/PROCHECK/) were used to validate the optimized 3D structure [36–39].

### In silico cloning optimization of vaccine construct

The Backtranseq server (https://www.ebi.ac.uk/Tools/st/emboss_backtranseq/) [40] was used to reverse the sequence translation of the designed vaccine structure, and expression in the host cell will be reduced owing to the lack of codon optimization; therefore, JCat server (http://www.jcat.de/) [41] was used to optimize the translation codon of *E. coli* K12 to optimize the codon structure of the multi-epitope vaccine. Finally, the construct sequence of the optimized multi-epitope vaccine was cloned into the pET28a (+) vector using The SnapGene program. Virtual agarose gel simulation was used to virtualize the clone.

### Molecular docking

Server ClusPro 2.0 (https://cluspro.org) [42] was used for protein–protein docking between HLA-A02:01 receptor and ligand (designed vaccine construct). This server fulfilled the task in triple continuous steps like rigid body docking, clustering of lowest form of energy and structural refinement by energy minimization [43]. The best-docked complex was picked according to the minimum energy scoring and docking effectiveness.

### Molecular dynamics simulation

Molecular dynamics is a computational method that was conducted to demonstrate the behavior of molecules and to evaluate the stability of protein–protein complexes [44]. In this study, iMODS server was used to explore the interactivity of the created vaccine and its receptor as it has the merit of rapidness and high efficacy [45]. This server evaluates the trend and span of the basic movements of the protein–ligand compound through assessing four prominent reasons: B-factors, eigenvalues, deformability and covariance. In general, when there is high eigenvalue, distortion is very harder [46].

### In silico evaluation of immune response

To evaluate the immunogenicity of the ultimate vaccine, in silico immune simulations were performed by utilizing the C-ImmSim server. This immune trigger applies a position-specific scoring matrix (PSSM) and machine learning methods in order to estimate epitope prediction and immune interactivities, respectively [47].

Clinically, the minimum period of time suggested between two doses of vaccines is 1 month [48]. Immune simulation was conducted by applying the identical protocol reported by previous studies [49, 50]. In brief, three inoculations were administered with the suggested periods of time of 1 month (1, 84 and 168 time steps variables were prepared, as one time step is similar to 8 hours of everyday life) for a total of 1050 steps of triggering. All other triggering parameters were kept as defaults.

## Results

### Prediction of B cell immune epitopes

The overlap results of the predicted linear B cell epitopes which were found by IEDB and ABCPred servers for proteins S and N are shown in Table 1.

**Table 1:** Overlap results of predicted linear B cell epitopes of IEDB and ABCPred servers of S and N proteins

S protein		N protein	
Position	Peptide sequence	Position	Peptide sequence
329	FPNITNLCPFGEVFNA	249	KSAAEASKKPRQKRTA
374	FASVYAWNRKRISNCV	24	TGSNQNGERSGARSKQ
674	YQTQTNSPRRARSVAS	59	HGKEDLKFPRGQGVPI
1153	DKYFKNHTSPDVDLGD	182	ASSRSSSRSRNSSRNS
19	TTRTQLPPAYTNSFTR	376	ADETQALPQRQKKQQT
1139	DPLQPELDSFKEELDK	77	NSSPDDQIGYYRRATR
374	FSTFKCYGVSPTKLND	362	TFPPTEPKKDKKKKAD
1146	DSFKEELDKYFKNHTS	243	GQTVTKKSAAEASKKP
250	TPGDSSSGWTA	237	KGQQQQGQTVTKKSAA
786	KQIYKTPPIKDFGGF	32	RSGARSKQRRPQGLPN
659	YTMSLGAENSVAYSNN	169	KGFYAEGSRGGSQASS
484	EGFNCYFPLQSYGFQP	71	GVPINTNSSPDDQIGY
445	VGGNYNYLYRLFRKSN	200	GSSRGTSPARMAGNGG
457	RKSNLKPFERDISTEI	387	KKQQTVTLLPAADLDD
177	MDLEGKQGNFKNL	143	KDHIGTRNPANNAAIV
293	LDPL	65	KFPRGQGVPINTNSSP
555	SNKKFLPF	192	NSSRNSTPGSSRGTSP
580	QTLE	266	RLNQLESKMSGKGQQQ
602	TNTSN	4	NGPQNQRNAPRI
634	RVYSTGSNVFQ	119	AGLPYGANK
695	YTMSLGAENSVAYSNN	343	DPNFKD
773	EQDKNTQ	404	SKQLQQSMSSADS
807	PDPSKPSK		
988	EAEVQ		
1035	GQSKRVDFC		
1107	RNFYEPQIITTD		

### Prediction of cytotoxic T lymphocyte and HTL epitopes

The prediction results of CTL epitopes (9 mer) with nHLAPred server (Table 2) and HTL epitopes (15 mer) with NetMHCIIpan 4.0 server for both S and N proteins are shown in Table 3.

**Table 2:** Results of CTL S and N protein epitope prediction by nHLApred server

S protein		N protein	
Position	Peptide sequence	Position	Peptide sequence
6	VLLPLVSSQ	222	LLLDRLNQL
7	LLPLVSSQC	316	GMSRIGMEV
109	TLDSKTQSL	331	LTYTGAIKL
119	IVNNATNVV	406	QLQQSMSSA
151	SWMESEFRV	48	NTASWFTAL
241	LLALHRSYL	226	RLNQLESKM
269	YLQPRTFLL	215	GDAALALLL
502	GVGYQPYRV	393	TLLPAADLD
512	VLSFELLHA	138	ALNTPKDHI
24	LPPAYTNSF	45	LPNNTASWF
56	LPFFSNVTW	66	FPRGQGVPI
84	LPFNDGVYF	105	SPRWYFYYL
208	TPINLVRDL	222	LLLDRLNQL
241	LLALHRSYL	278	GPEQTQGNF
250	TPGDSSSGW	395	LPAADLDDF
321	QPTESIVRF	219	LALLLLDRL
478	TPCNGVEGF	312	SAFFGMSRI
704	SVAYSNNSI	111	YYLGTGPEA

**Table 3:** HTL epitope prediction results by NetMHCIIpan 4.0 server

S protein		N protein	
Position	Peptide sequence	Position	Peptide sequence
867	DEMIAQYTSALLAGT	388	KQQTVTLLPAADLDD
309	EKGIYQTSNFRVQPT	168	PKGFYAEGSRGGSQA
430	TGCVIAWNSNNLDSK	374	KDQVILLNKHIDAYK
232	GINITRFQTLLALHR	311	ASAFFGMSRIGMEVT
512	VLSFELLHAPATVCG	354	NKHIDAYKTFPPTEP
59	FSNVTWFHAIHVSGT		
323	TESIVRFPNITNLCP		
1001	LQSLQTYVTQQLIRA		
314	QTSNFRVQPTESIVR		
790	KTPPIKDFGGFNFSQ		
978	NDILSRLDKVEAEVQ		
540	NFNFNGLTGTGVLTE		

### Selection of epitopes and design of vaccine structures

**Fig. 1:**
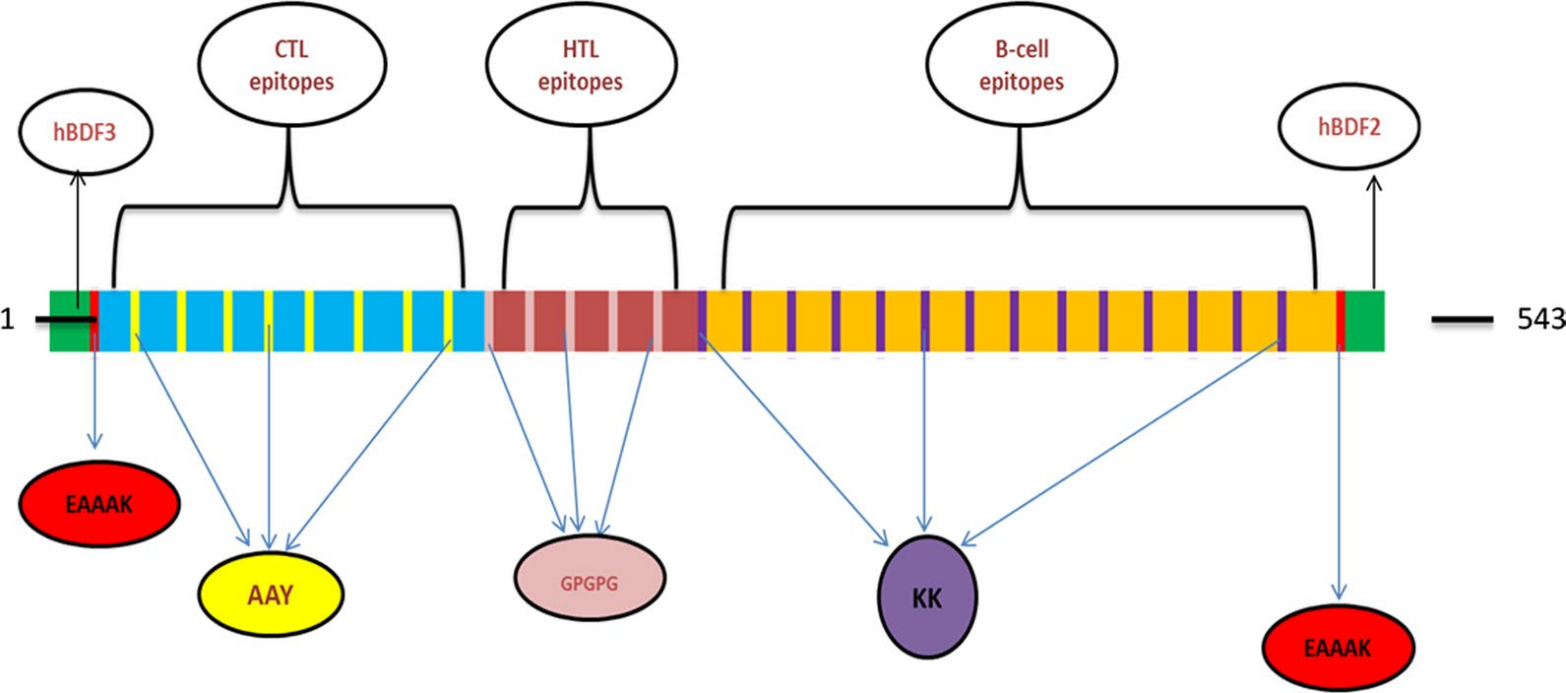
Schematic specifications of the structure of a 543-amino-acid-long multi-epitope vaccine. Adjuvant with EAAAK linker is added to the beginning and end of the structure, and CTL, HTL and B cell epitopes are connected with AAY, GPGPG and KK linkers, respectively

The accuracy of the epitopes predicted for B cell, CTL and HTL, in terms of allergenicity, antigenicity and toxicity, was examined. In order to design the vaccine structure, both non-allergenic and non-toxic epitopes that had antigenic potency were selected. Finally, 9 CTL epitopes (5 epitopes for N protein and 4 epitopes for S protein), 5 HTL epitopes (1 epitope for N protein, 4 epitopes for S protein (Table 4) and 14 B cell epitopes (11 epitopes for N protein and 3 epitopes for S protein) (Table 5) were selected for vaccine structure design. The selected CTL, HTL and B cell epitopes were connected by AAY, GPGPG and KK linkers, respectively. As adjuvant, hBD-3 with 45 amino acids and hBD-2 with 41 amino acids were added to the N and C ends of the structure with EAAAK linker. The final vaccine construct consisted of 543 amino acids (Fig. 1).

**Table 4:** Selected CTL and HTL epitopes of S and N proteins for vaccine structure design

	N protein					S protein				
	Peptide sequence	Position	Allergenicity	Toxicity	VaxiJen score	Peptide sequence	Position	Allergenicity	Toxicity	VaxiJen score
CTL epitopes	GMSRIGMEV	316	NON-ALLERGEN	Non-Toxin	0.6287	TLDSKTQSL	109	NON-ALLERGEN	Non-Toxin	1.0685
	GDAALALLL	215	NON-ALLERGEN	Non-Toxin	0.4529	GVGYQPYRV	502	NON-ALLERGEN	Non-Toxin	1.3587
	SPRWYFYYL	105	NON-ALLERGEN	Non-Toxin	0.7340	LPFNDGVYF	84	NON-ALLERGEN	Non-Toxin	0.5593
	GPEQTQGNF	278	NON-ALLERGEN	Non-Toxin	0.7349	**VLSFELLH**	512	NON-ALLERGEN	Non-Toxin	1.0776
	LALLLLDRL	219	NON-ALLERGEN	Non-Toxin	0.5933					
HTL epitopes	KQQTVTLLPAADLDD	388	NON-ALLERGEN	Non-Toxin	0.6264	GINITRFQTLLALHR	232	NON-ALLERGEN	Non-Toxin	0.5582
						**VLSFELLH**APATVCG	512	NON-ALLERGEN	Non-Toxin	0.4784
						FSNVTWFHAIHVSGT	59	NON-ALLERGEN	Non-Toxin	0.7533
						TESIVRFPNITNLCP	323	NON-ALLERGEN	Non-Toxin	0.6128

Overlapping sequence of CTL and HTL epitopes sequences was bolded

**Table 5:** Selected linear epitopes of B cell, S and N proteins for vaccine structure design

	N protein					S protein				
	Peptide sequence	Position	Allergenicity	Toxicity	VaxiJen score	Peptide sequence	Position	Allergenicity	Toxicity	VaxiJen score
B cell epitopes	DPNFKD	343	NON-ALLERGEN	Non-Toxin	2.878	FSTFKCYGVSPTKLND	374	NON-ALLERGEN	Non-Toxin	0.965
	RLNQLESKMSGKGQQQ	266	NON-ALLERGEN	Non-Toxin	1.0049	DSFKEELDKYFKNHTS	1146	NON-ALLERGEN	Non-Toxin	0.965
	RSGARSKQRRPQGLPN	32	NON-ALLERGEN	Non-Toxin	0.7271	DKYFKNHTSPDVDLGD	1153	NON-ALLERGEN	Non-Toxin	0.614
	ADETQALPQRQKKQQT	376	NON-ALLERGEN	Non-Toxin	0.6949					
	GQTVTKKSAAEASKKP	243	NON-ALLERGEN	Non-Toxin	0.6491					
	TGSNQNGERSGARSKQ	24	NON-ALLERGEN	Non-Toxin	0.6333					
	HGKEDLKFPRGQGVPI	59	NON-ALLERGEN	Non-Toxin	0.6163					
	TFPPTEPKKDKKKKAD	362	NON-ALLERGEN	Non-Toxin	0.5442					
	GSSRGTSPARMAGNGG	200	NON-ALLERGEN	Non-Toxin	0.5625					
	KSAAEASKKPRQKRTA	249	NON-ALLERGEN	Non-Toxin	0.4636					
	NSSRNSTPGSSRGTSP	192	NON-ALLERGEN	Non-Toxin	0.4904

### Predicting the next three structures and optimizing the vaccine structure

**Fig. 2:**
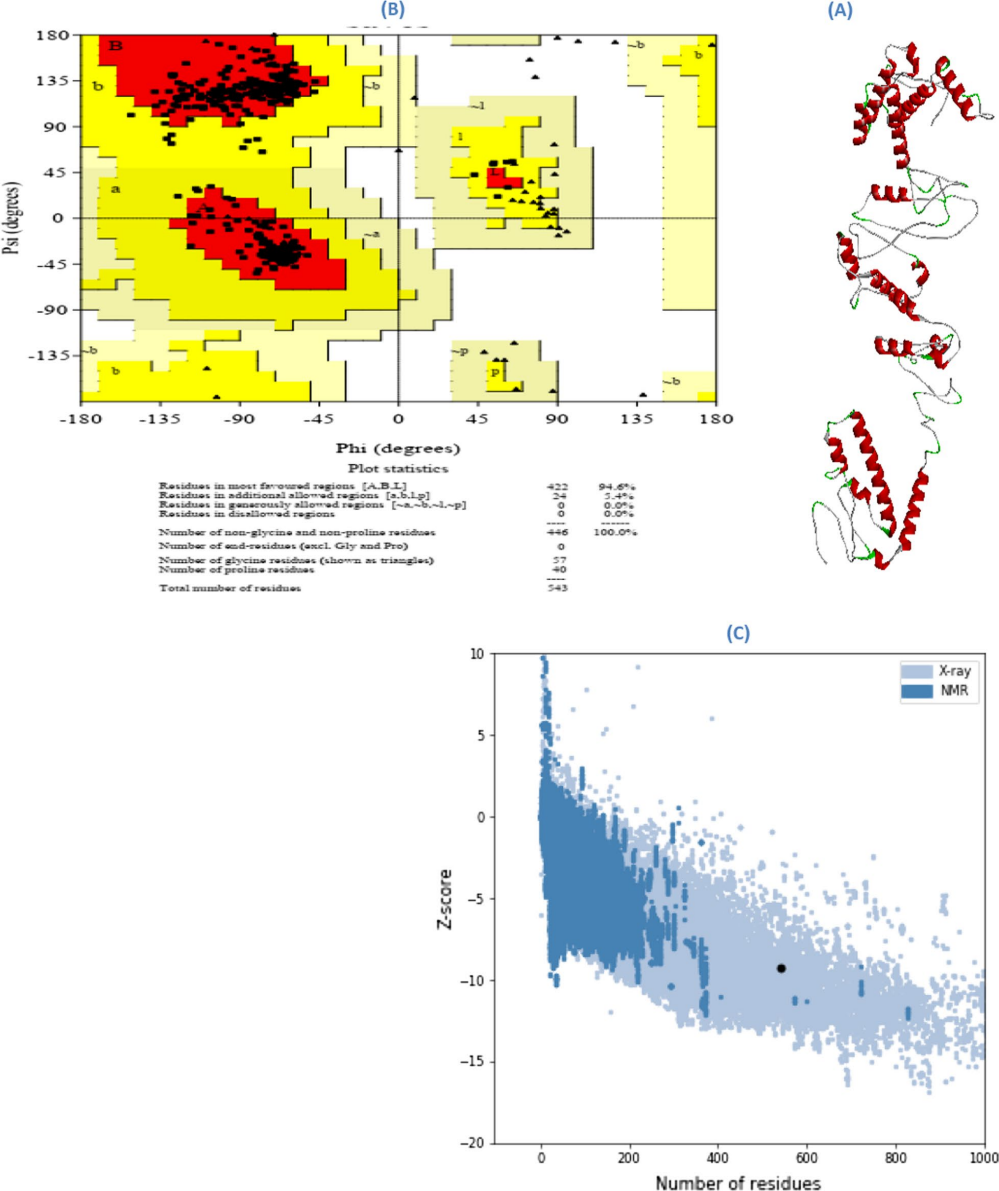
Evaluation of the third optimized structure of the vaccine structure. **a** Optimized three-dimensional structure of the vaccine structure, **b** analysis of the vaccine structure by Ramachandran map, **c** Z-score map of ProSA server vaccine structure

Phyre2, RaptorX and I-TASSER were used in order to predict the three-dimensional structure of different servers, which were evaluated by Ramachandran map. After reviewing the characteristics and validity of the predicted structures by using different servers, the structure received from the RaptorX server, which had a better quality than other servers, was selected. GalaxyRefine server was used to optimize the selected 3D structure. Out of five optimization models, model number 4 with higher RMSD and GDT-HA was selected (Fig. 2a and Additional file 1).

### Analysis of stereochemical properties and prediction of solubility, allergenicity, toxicity and antigenicity of vaccine structures

Examination of the stereochemical properties of the designed structure using ProtParam program showed that the molecular weight of the vaccine structure is 59038.88 daltons with an isoelectric point of 10.06 which shows the basic nature of the designed vaccine structure. The total number of negatively charged amino acids (glutamic, aspartic acid) is 39, and the total number of positively charged amino acids (arginine, lysine) is 104. The aliphatic index is 57.24 and the instability of the designed structure was reported to be 38.96, which indicates the stability of the vaccine structure designed in the host. GRAVY index was − 0.7, and it was reported that the negative of this index indicates that the vaccine structure is hydrophilic, so it could interact well with water molecules. The half-life of this vaccine construct was predicted to be 30 h in mammals (in vivo), more than 20 h in yeast (in vivo) and more than 10 h in *E. coli* (in vivo). Based on the results of the SOLpro server, the designed vaccine structure was predicted soluble with a probability of 0.9, which ensures easy access to the host. Also designed vaccine structures were predicted to be non-toxic, non-allergenic and antigenic. The result of its antigenicity according to VaxiJen and ANTIGENpro servers is 0.5 and 0.9, respectively.

### Features of the secondary predicted structure

**Fig. 3:**
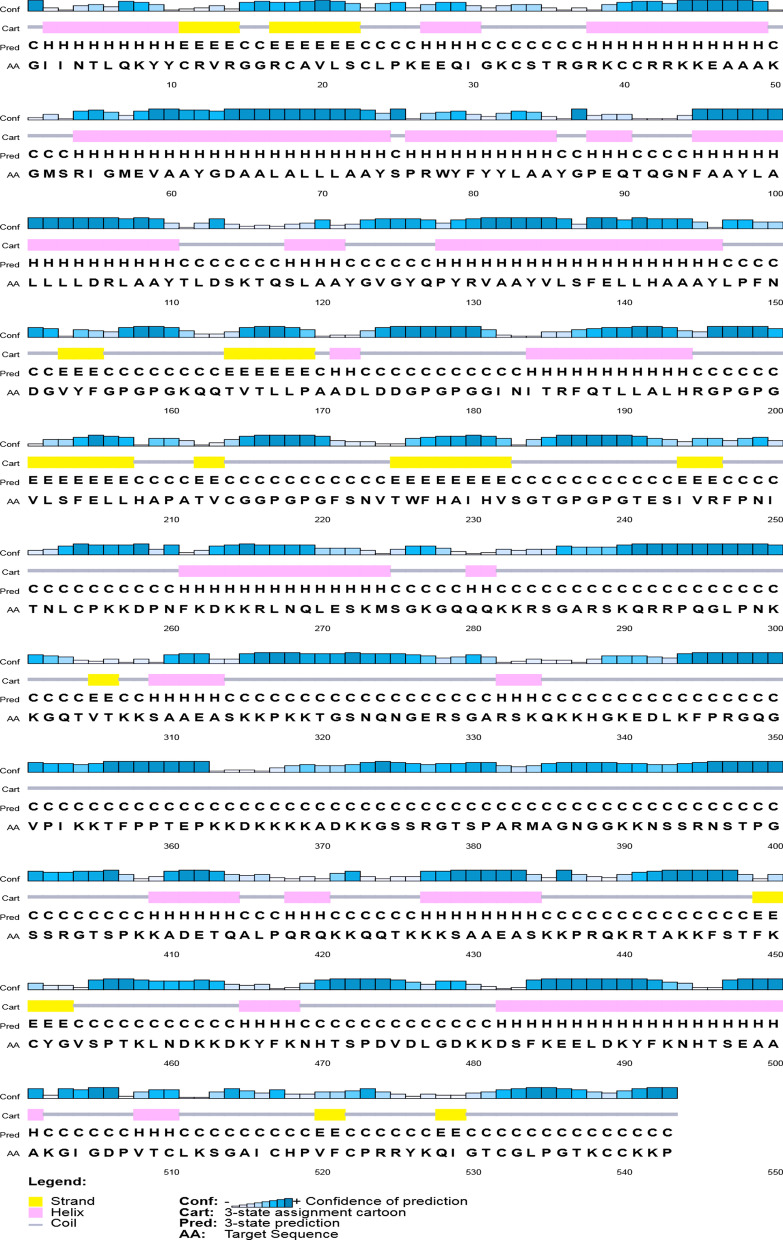
Two-dimensional analysis of the structure of the vaccine designed by the PSIPRED server

The second structure of the protein using the PSIPRED program is shown in Fig. 3. Also, according to the results of SOPMA program, the protein has 145 alpha helices (26.70%), 84 extended strands (15.47%), 36 β-turn (6.63%) and 278 random coils (51.20%) (Additional file 1).

### Validation of the optimized three-dimensional structure of the vaccine structure

Structural validation is a procedure to recognize potential flaws in the estimated tertiary structure [51]. The overall quality assessment of the optimized 3D structure was evaluated with ProSA, ERRAT and PROCHECK servers. According to the results of ERRAT and ProSA servers, the quality factor was 92,000 and Z-score of the structure reported was − 9.29 (Additional file 1) which is in the range of scores that are normally found for natural proteins of similar size (Fig. 2c). Also, according to the Ramachandran map which was obtained from the PROCHECK server, the number of amino acids in favored and allowed regions is 94.6% and 5.4%, respectively, and in outlier regions, 0.0% was reported (Fig. 2b).

### Codon optimization and in silico cloning

**Fig. 4:**
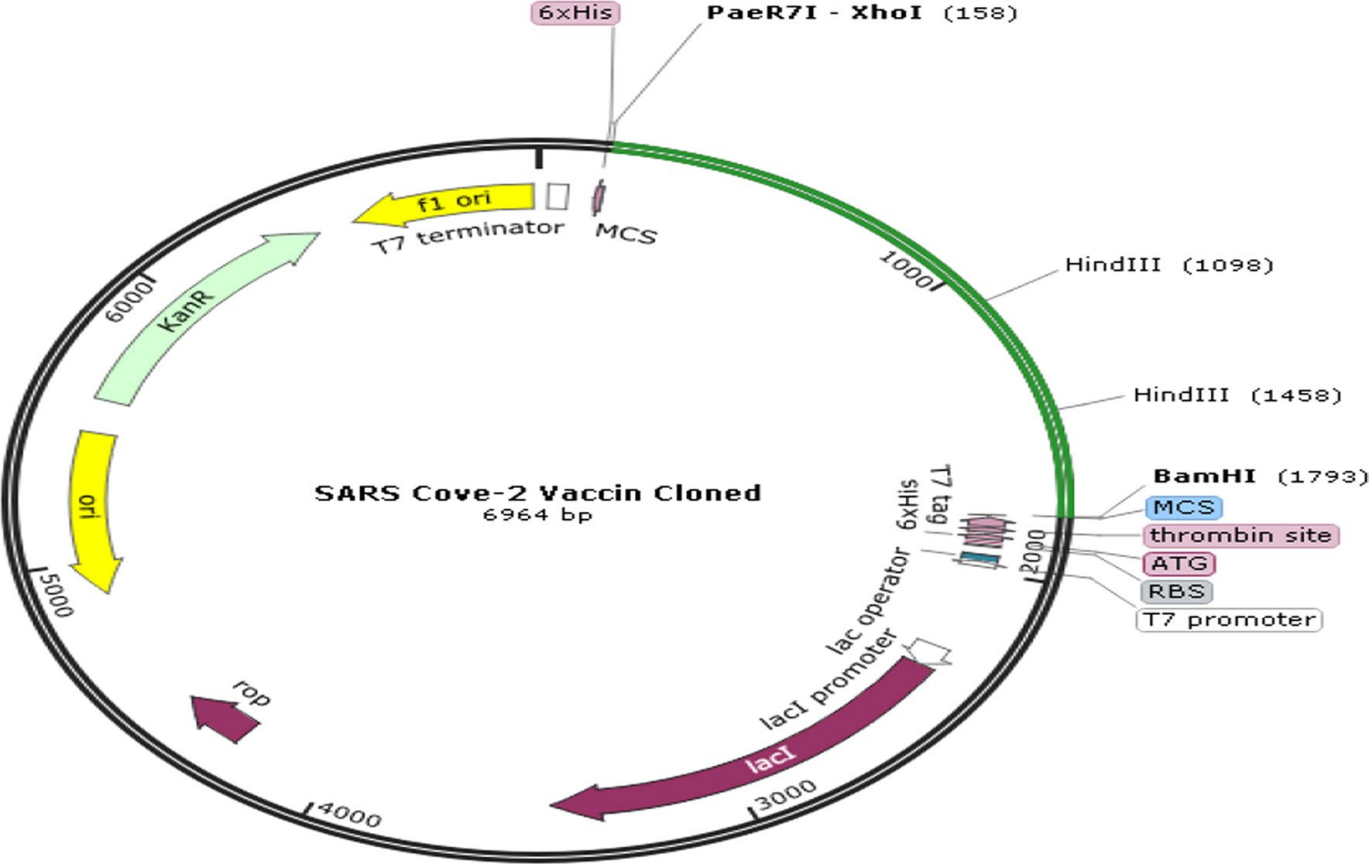
Clone of the designed vaccine construct. The optimized codon sequence of the designed vaccine construct (shown in green) was cloned between the XhoI and BamHI enzyme loci in the expression vector pET-28a (+) (shown in black)

**Fig. 5:**
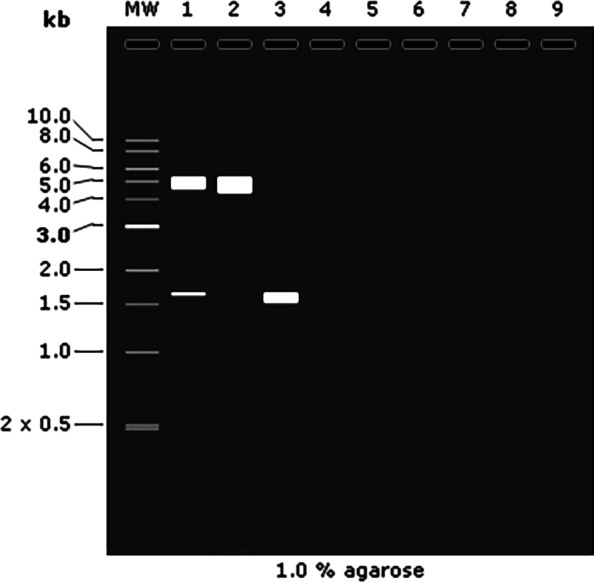
Virtual clone of a vaccine construct designed with dual digestion. Line1: Digestive structure of vaccine (vaccine and vector) with two enzymes XhoI and BamHI; line2: digestion of two enzymes, vector pET-28a (+); line 3: digestion of two enzymes, incert (designed vaccine)

In order to evaluate the cloning and expression of the vaccine construct in the expression vector, the inverse translation of the vaccine construct sequence was received by the Backtranseq server and its codon was optimized by the JCAT server. The JCAT server evaluates the sequence to optimize the codon and reports the codon compatibility index (CIA) and GC content of the sequence. According to the results, the codon compatibility index (1.0), which was in the optimal range (0.8–1.0), was calculated. A high CIA value indicates high gene expression. Also, GC sequence content (51.93%) was in the desired range (30–70%). These results may indicate high expression of the vaccine construct in the bacterial system. Finally, after adding the BamHI and XhoI restriction enzymes to the sequence, the optimized codon sequence was cloned using the SnapGene program in the pET28a (+) vector (Fig. 4). The SnapGene program virtual agarose gel simulation shows the presence of insert alone, along with vector after digestion with BamHI and XhoI enzymes (Fig. 5).

### Molecular docking

**Fig. 6:**
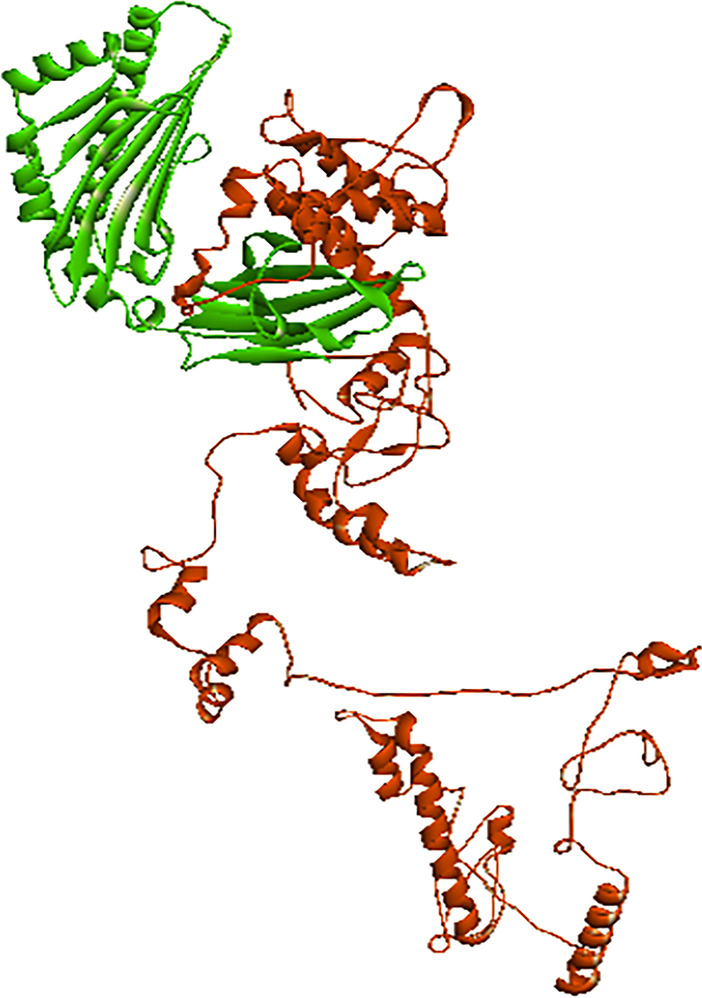
Docking complex of vaccine structure with A-chain HLA-A02:01 receptor. The receptor is shown in green, and the vaccine structure is shown in red

**Fig. 7:**
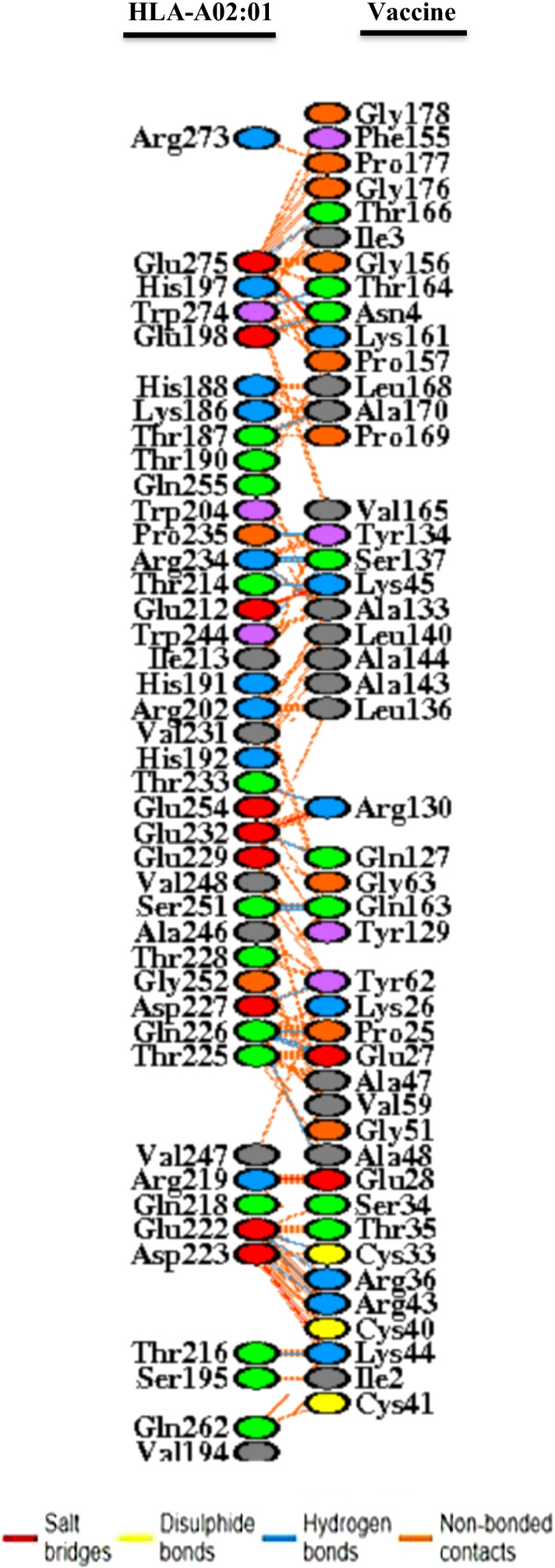
Interacting residues illustration between vaccine construct and HLA-A02:01: A total of 46 residues of the vaccine associated with 42 residues of the HLA-A02:01 molecular. A number of 25 hydrogen bonds (blue line) were formed between the residues of the HLA-A02:01 molecule and the residues of the vaccine

Docking of the designed vaccine construct as a ligand with HLA-A02;01 (PDB ID: 3TO2) as the receptor was performed by the ClusPro 2.0 server. This server predicts 30 complexes and classifies them based on the amount of energy. Among the predicted models, model 4, which had the lowest energy weighted score of − 1158.9, has been selected as the best model for vaccine interaction with HLA-A02:01 (Fig. 6). Additionally, PDBsum as a virtual database was applied to show the interacting residues of docked complexes [52]. An amount of 46 vaccine residues was matched with 42 residues of chain A from HLA-A02;01 molecule. Also, 25 hydrogen bonds were built between the residues of the chain A from the HLA-A02:01 molecule (Fig. 7).

### Molecular dynamics simulation

**Fig. 8:**
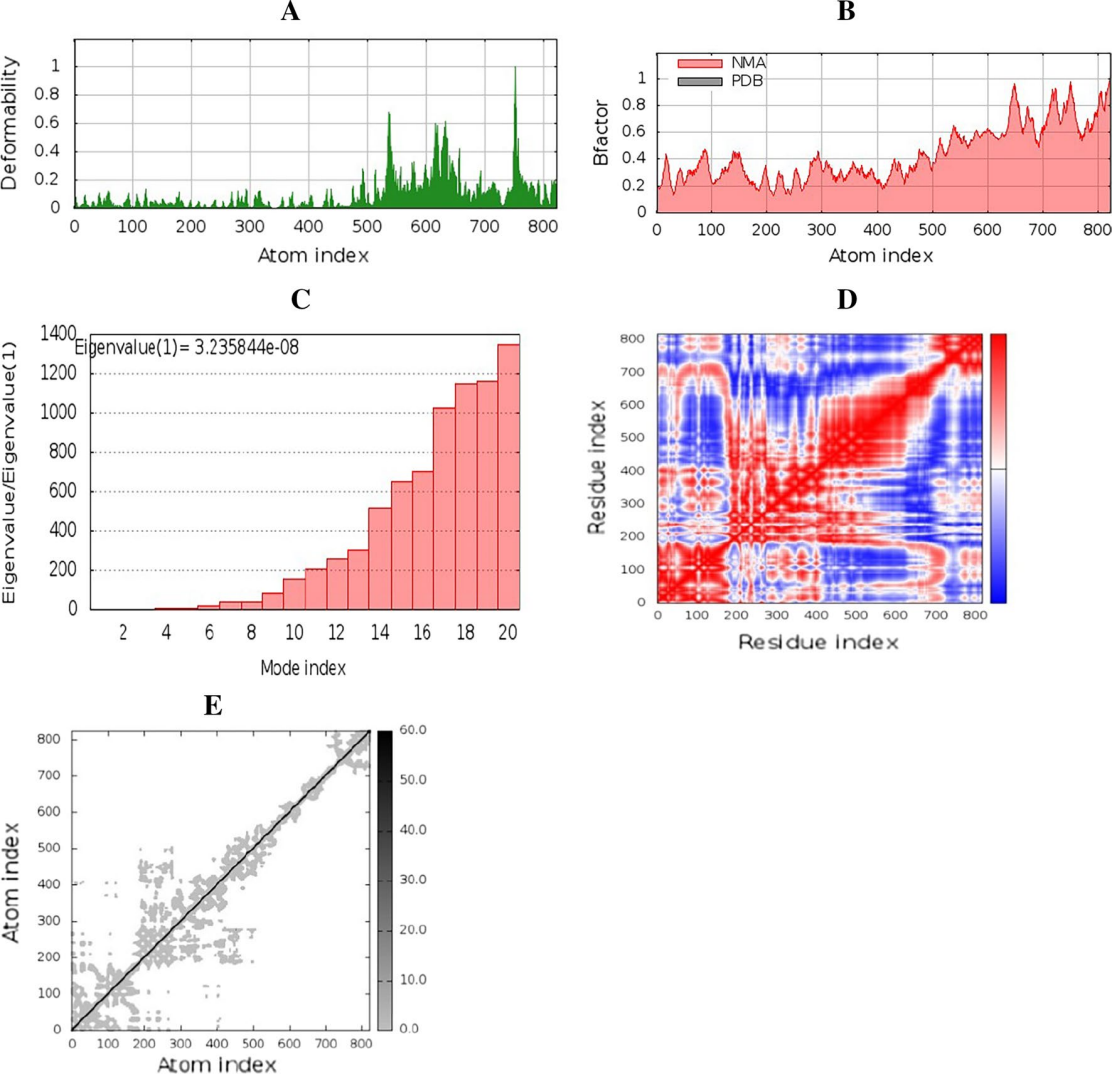
Molecular dynamics simulation of multi-epitope vaccine—HLA-A02:01 complex; stability of the protein–protein complex was investigated through deformability (**A**), B-factor values (**B**), eigenvalue (**C**), covariance of residue index (**D**) and elastic network (**E**) analysis

To assess the firmness and physical motions of the created vaccine composition—HLA-A02:01 docked compound. Molecular dynamics simulation was performed through the iMOD server [46]. The main chain deformability is displayed in Fig. 8A. The region where hinges are located has a high tendency to deform. The B-factor values computed by normal mode analysis are proportional to root mean square (Fig. 8B). Values of B-factor measure the unpredictability of each atom. Figure 8C introduces the eigenvalues having close correlation with the energy needed to distort the formation. The eigenvalue of the complex is 3.23e^−08^. The covariance matrix between the pairs of residues is displayed in Fig. 8D, showing their correlations (red: correlated, white: uncorrelated, blue: anti-correlated). The elastic network model is indicated in Fig. 8E.

### In silico evaluation of immune response

**Fig. 9:**
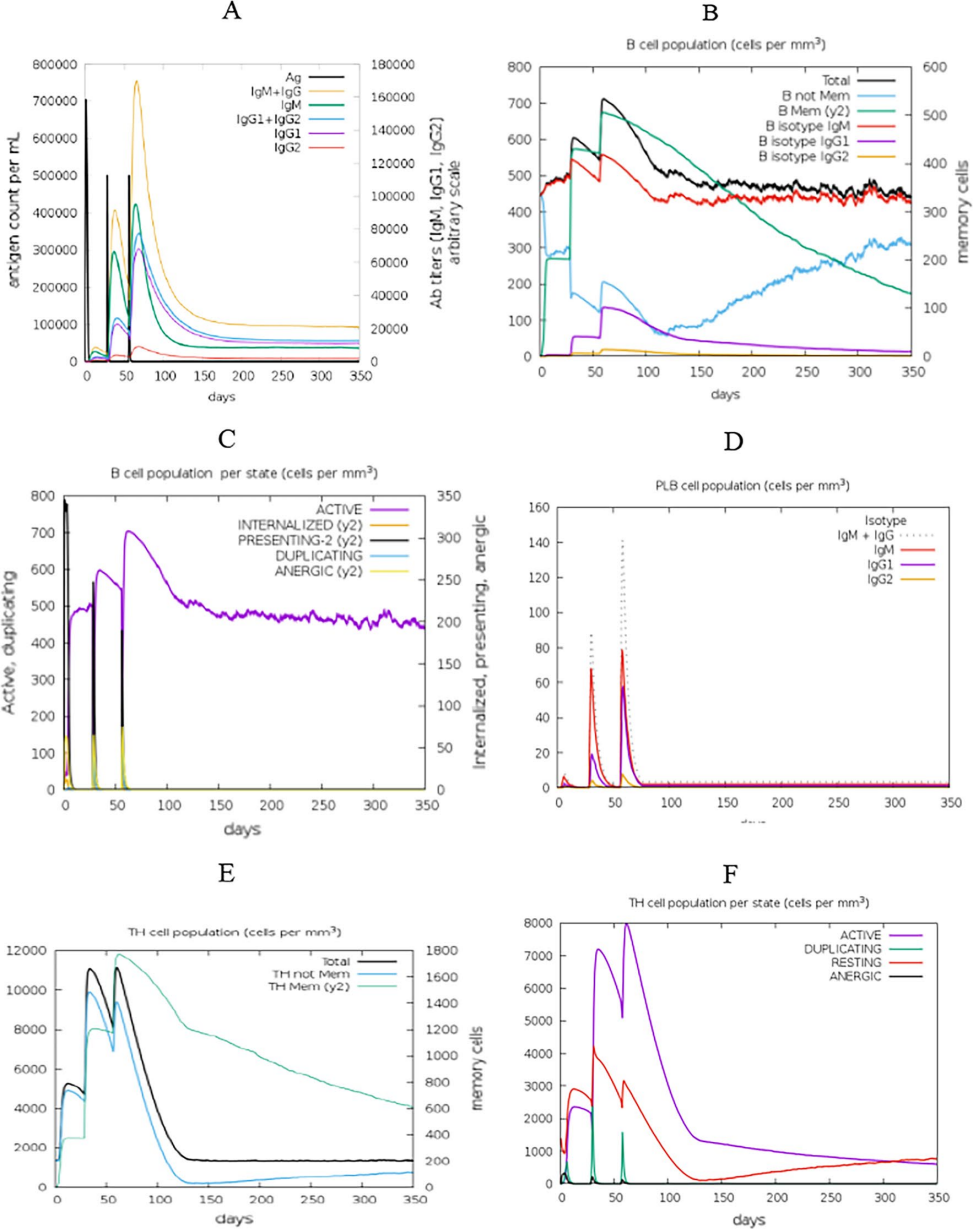

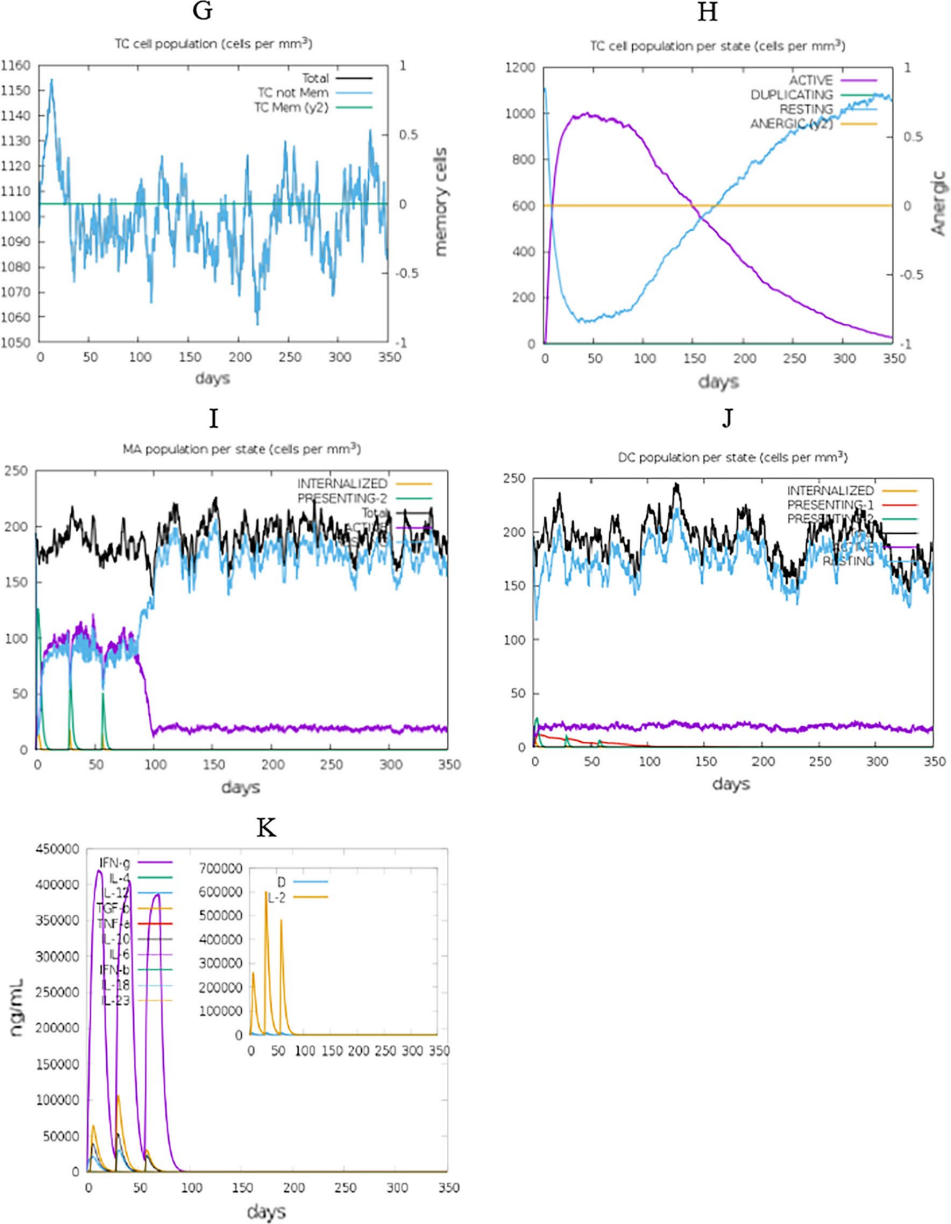
The immune simulation results of the vaccine construct. **A** Immunoglobulins levels with respect to antigen concentration, **B** B cell population, **C** B cell population per state, **D** plasma B cell population, **E** helper T cell population, **F** helper T cell population per state, **G** cytotoxic T cell population, **H** cytotoxic T cell population per state, **I** macrophage population per state, **J** dendritic cell population per state and **K** production of cytokine and interleukins with Simpson index

The immunogenic profile of the designed vaccine candidates was attained from C-IMMSIM server. Simulation outcomes depicted that high concentrations of IgM were recognized at the primary response. In both secondary and tertiary responses, the usual elevated levels of immunoglobulin activities (i.e., IgG1 + IgG2, IgM, and IgG + IgM antibodies) were noticeable with associated antigen depletion (Fig. 9A). The elevated levels of simulated B cells and memory B cell formation were seen, which shows a productive long-established immune reaction created by the vaccine structure (Fig. 9B–D). A further high level of reaction was seen in the T helper and cytotoxic T cell populations with relative memory establishment which is necessary to trigger the immune reaction (Fig. 9E–H). Thus, improved activity of macrophage was observed while dendritic cell activity was steady (Fig. 9I, J). It was also found high level of cytokines including IFN-γ and IL-2, which are imperant for inhibition of viral replication and cellular immunity (Fig. 9K). The above-observed immune elicit characteristics ensured that vaccine structure would be effectual in human subjects.

## Discussion

Today, as coronaviruses appear periodically and unpredictably and they are spreading rapidly, they are causing serious infectious diseases; they have become a constant threat to human health. This is especially true when there is no vaccine or approved drug to treat coronavirus infection [1]. Many studies are underway to develop an effective vaccine against SARS-CoV-2. Some studies have suggested that the S protein is a promising candidate for the SARS-CoV-2 vaccine because it is involved in the binding, fusion and entry of the virus into the host cell [53]. There are also reports showing that antibodies against S protein prevent SARS-CoV-2 from entering cells, so it strengthens the use of S protein as a suitable candidate for the production of SARS-CoV-2 vaccine [54]. Also, N protein, due to its protected protein sequence, growing knowledge of its genetic biochemistry and very high immunogenicity, can be considered as a suitable candidate for the production of vaccine against COVID-19 disease [55]. Today, the ease of manufacturing industrial peptides as well as their engineering ability has made such vaccines suitable candidates for vaccination. The use of epitope vaccines based on peptide synthesis is one of the new strategies in vaccine research that focuses the immune response on important and valuable epitopes. The use of epitope peptides for vaccination against various organisms such as HIV (human immunodeficiency virus), HBV (hepatitis B virus) and various models of cancer, etc., has been considered [56]. During the present study, epitopes derived from S and N proteins SARS-CoV-2 were studied for the design and development of a multi-epitope vaccine using immunoinformatic methods. Identification of antigenic epitopes by the immune system is a key step in the immune response to the pathogen, identifying either epitopes that stimulate T cells or epitopes that are trapped by B cells and soluble antibodies [57]. In this study, CTL, HTL and B cell epitopes were selected based on antigenetic, allergenicity and toxicity. A restriction of these published studies is the failure to consider the effect of glycosylation, which could shield some of the selected epitopes. The vital role of glycosylation is defined in antigenicity, fusogenic and immunomodulatory activities of the spike protein [58]. About 17 N-glycosylation sites associated with two O-glycosylation sites were found occupied in the spike protein of SARS-CoV-2 [59]. Meanwhile, glycans could impede the recognition of antigens by shielding the residues [60], and protein glycosylation would impact on the efficiency of antigen finding [61]. We circumnavigated most glycosylation sites when selecting epitopes derived from S protein SARS-CoV-2. In this study, only three selected epitopes (GI**N**^**234**^**ITR**FQTLLALHR, FS**N**^**61**^**VTW**FHAIHVSGT, TESIVRFP**N**^**331**^**ITN**LCP) contain glycosylation sites, which should have a minimum influence on antigen recognition. If these glycosylation sites hinder the diagnostic presentation, an extra deglycosylation step with *N*-glycanase should be useful for the test samples, which is a simple and useful technique for deglycosylation [61]. Many studies have revealed the influence of glycosylation on the augmentation of antigens immunogenicity [62]. Owing to increase expression, folding and stability, linkers act as an essential element in the development of epitope vaccines [63]. In this study, CTL, HTL and B cell epitopes were connected to design vaccine structure by AAY, GPGPG and KK linkers, respectively. Defensins increase the acquired immune response by chemically absorbing activity for monocytes, T cells and dendritic cells, and the activity of inducing cytokine production by monocytes and epithelial cells [64]. Accordingly, human beta-defensin 3 and 2 were added as adjuvants to the N and C ends of the designed structure by the EAAAK linker, respectively. EAAAK linker, due to its salt bridge related to glutamic acid and lysine, can prevent protein which domains from converging by creating a stable helix structure [65].

The molecular weight of the designed vaccine was 59,038.88 daltons (approximately 59 kDa), which makes it an acceptable vaccine. Because proteins with a molecular weight of less than 110 kDa are considered as more suitable targets for vaccine production [66]. The isoelectric point of the vaccine structure was determined to be 10/06, which indicates the playful nature of the designed vaccine structure. Also, the instability index of the structure is 38.96 according to ProtParam program, which is classified as a stable protein. Because the range of this index for stable proteins is less than 40 results, the alpha index, which indicates the stability of the protein over a wide temperature range, was reported to be 57.24 for this designed vaccine construct. Its GRAVY value is − 0.7, which is a negative value of this index, indicating the nature of the hydrophilic structure of the vaccine, and therefore can interact strongly with water molecules. The total number of negatively charged (Asp + Glu) and positive (Arg + Lys) amino acids in this vaccine structure is 39 and 104, respectively. The half-life of this vaccine construct was predicted to be 30 h in mammals, more than 20 h in yeast and more than 10 h in *E. coli*. Based on the results, the structure of the designed vaccine solution was predicted to ensure easy access to the host. Also, according to the predicted results, the structure of the designed vaccine is antigen, non-toxic and non-allergenic. The quality of the three-dimensional structure of the designed vaccine structure increased dramatically after optimization, so that all amino acids in the desired and allowed areas (100%), according to Ramachandran map, reported that it shows the appropriate quality of the three-dimensional structure of the designed vaccine structure. Various tools were used to determine possible errors and the quality of the three-dimensional structure of the designed vaccine structure. Z-score (− 9.22) and ERRAT quality factor (92,000) showed that the structure of the designed vaccine is appropriate. Using ClusPro2.0 server, connection was made between the vaccine structure designed with HLA-A02:01—1158.9 of HLA-A02:01 was the lowest amount of energy in the total of the vaccine structure. Furthermore, the iMODS server was applied to evaluate the constructional steadiness and atomic-level motions of docked complex (designed vaccine construct—HLA-A02:01). It showed that docked proteins have minor deformation for each residue and with establishing our estimation of eigenvalues for 3.23e^−08^, which display the validity of our in silico predicted vaccine. Because all codons that are synonymous in a codon family do not use the same rate of expression of heterogeneous proteins in *Escherichia coli*, codon optimization in production of eukaryotic proteins is necessary in prokaryotic hosts [67]; therefore, codon optimization was performed to achieve a high level of protein expression in *E. coli* K12, and according to the results, both codon compatibility index (1.0) and GC percentage (51.93%) were calculated; this reveals a high probability of protein expression in bacteria. In addition, the immune simulation of the designed vaccine structure showed hopeful results regarding both humoral and cellular immune reaction. The results of bioinformatics evaluation of the designed vaccine construct indicated that this vaccine candidate may be highly potent against SARS-CoV-2, but in vitro and in vivo studies are needed for clinical confirmation.

## Conclusion

In silico vaccine formation being efficient is substantially important, and it strongly focused on the multi-epitope peptides of the vaccine. In this study, using bioinformatics analyses, suitable epitopes of S and N proteins were selected and analyzed. Finally, a different multi-epitope vaccine with a span of 543aa against the 2019-nCov will be created.

It consists of two adjuvants, with 14 B cell epitopes, 9 CTL epitopes and 5 HTL epitopes. It displays good antigenic features, immunological qualities and satisfactory physiochemical characteristics, non-allergenicity and non-toxicity. It is expected that the epitopes predicted in this study would be an efficient vaccine formation against COVID-19. However, the confirmation of the epitopes which were selected in this study as a vaccine candidate should be considered as laboratory studies.

## Supplementary Information

**Additional file 1**. Design of a multi-epitope-based peptide vaccine against the S and N proteins of SARS -COV-2 using Immunoinformatics Approach. (http://galaxy.seoklab.org/cgi-bin/report_REFINE.cgi?key=27ac3cd2f0bd1f0372ce673a67eac9e1, https://npsa-prabi.ibcp.fr/cgi-bin/secpred_sopma.pl, https://prosa.services.came.sbg.ac.at/prosa.php, https://saves.mbi.ucla.edu/results?job=748446&p=errat).

## Data Availability

URL links of supplementary files are available in Additional file 1.

## Abbreviations

Hbd2: Human β-defensin-2
Hbd3: Human β-defensin-3
2019-nCoV: 2019 novel coronavirus
SARS-CoV: Severe acute respiratory syndrome coronavirus
MERS: Middle East respiratory syndrome
SARS: Severe acute respiratory syndrome
MHC: Major histocompatibility complex
HLA: Human leukocyte antigen
pI: Isoelectric point
CTL: Cytotoxic T lymphocyte
HTL: Helper T lymphocyte
COVID-19: Coronavirus disease 2019
GRAVY: Grand average of hydropathy
pI: Isoelectric point
SARS-CoV-2: Severe acute respiratory syndrome coronavirus 2
