# Supplementary material for: Design of a multi-epitope-based peptide vaccine against the S and N proteins of SARS-COV-2 using immunoinformatics approach
Source: Egypt J Med Hum Genet. 2022 Feb 4;23(1):16. doi: 10.1186/s43042-022-00224-w (PMC8813187; doi:10.1186/s43042-022-00224-w)
Supplement: Supplementary file 1 — Additional file 1. Design of a multi-epitope-based peptide vaccine against the S and N proteins of SARS -COV-2 using Immunoinformatics Approach. (http://galaxy.seoklab.org/cgi-bin/report_REFINE.cgi?key=27ac3cd2f0bd1f0372ce673a67eac9e1, https://npsa-prabi.ibcp.fr/cgi-bin/secpred_sopma.pl, https://prosa.services.came.sbg.ac.at/prosa.php, https://saves.mbi.ucla.edu/results?job=748446&p=errat). [file 43042_2022_224_MOESM1_ESM.docx]

**Additional files:** Additional file 1 of Design of a multi-epitope based peptide vaccine against the S and N proteins of SARS -COV-2 using Immunoinformatics Approach

**ST**

| Model | GDT-HA | RMSD | Molprobity | Clash score | Poor rotamers | Rama favored |
| --- | --- | --- | --- | --- | --- | --- |
| Initial | 1.0000 | 0.0000 | 4.716 | 247.8 | 88.1 | 95.0 |
| MODEL1 | 0.9360 | 0.464 | 1.600 | 9.9 | 0.2 | 97.6 |
| MODEL2 | 0.9309 | 0.471 | 1.467 | 8.7 | 0.0 | 98.9 |
| MODEL3 | 0.9194 | 0.498 | 1.556 | 9.6 | 0.2 | 97.8 |
| MODEL4 | 0.9296 | 0.497 | 1.467 | 8.7 | 0.5 | 98.5 |
| MODEL5 | 0.9415 | 0.452 | 1.487 | 9.2 | 0.7 | 98.3 |


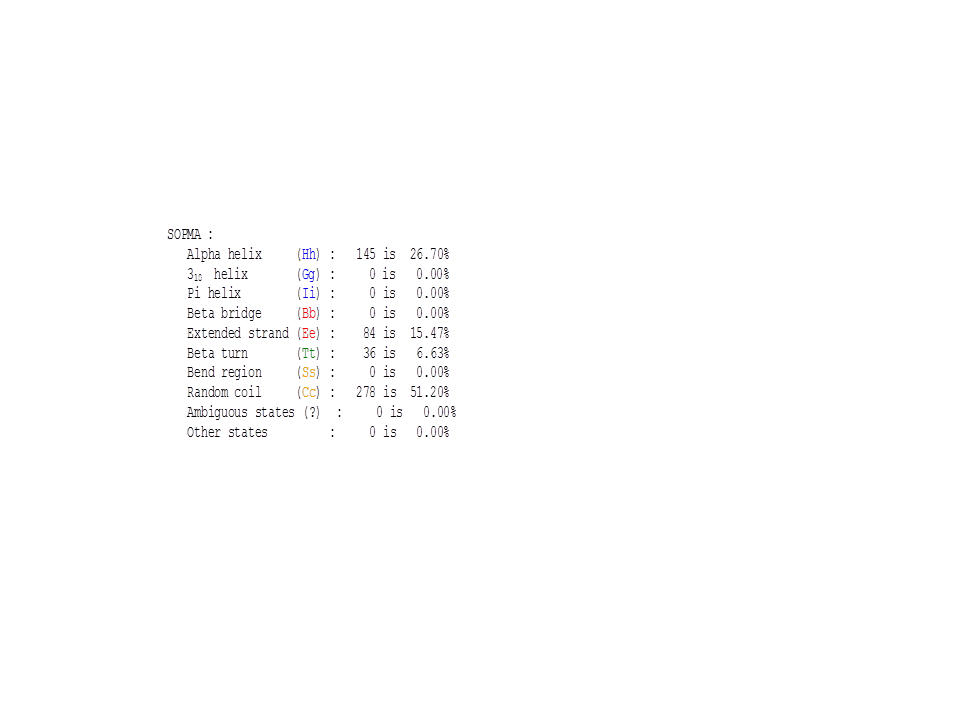


**SF1**

**A**


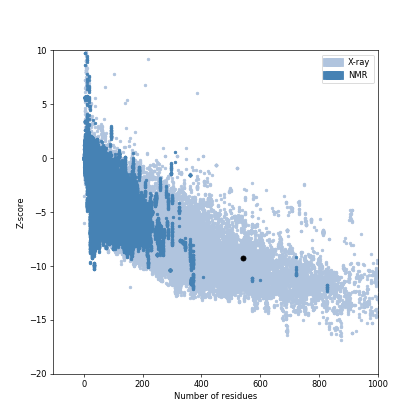


**B**


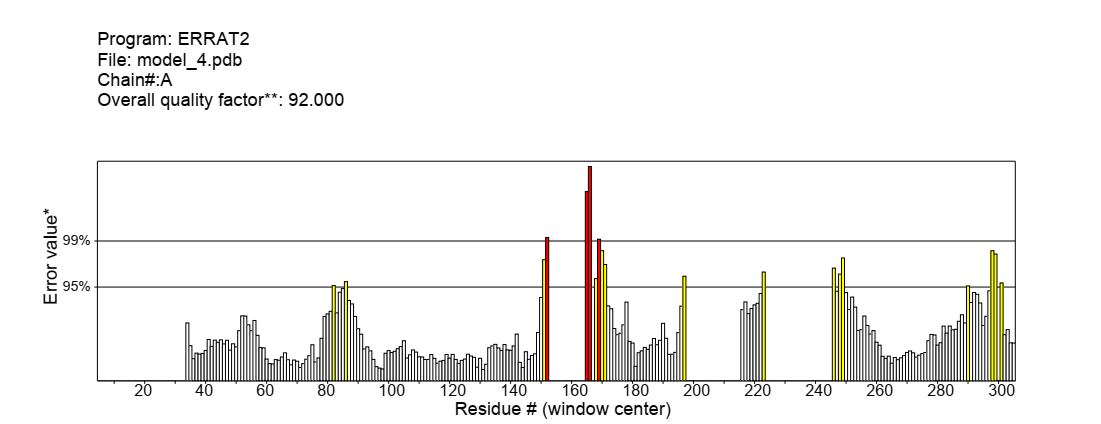


SF3

**Additional file 1**: ST,SF1,SF3
